# Effect of microplastic particles on the population growth rate and clearance rate of selected ciliates (Protista, Ciliophora)

**DOI:** 10.1007/s11356-023-31635-w

**Published:** 2023-12-29

**Authors:** Martyna Budziak, Janusz Fyda

**Affiliations:** Institute of Environmental Sciences, Jagiellonian University in Kraków, Gronostajowa 7, 30-387 Kraków, Poland

**Keywords:** Microplastics, Ciliates, Grazing, Population growth rate, Clearance rate

## Abstract

Microplastics (MPs), due to their micro size, which overlaps with the typical food size of various aquatic organisms, can be ingested and move up the food chain, accumulating in the bodies of organisms at higher trophic levels. Few studies have focused on the uptake of MPs by ciliates, which are an important element of the microbial cycle. Three different ciliate species were used in this study: *Blepharisma japonicum*, *Euplotes* sp., and *Spirostomum teres*, as well as polystyrene beads with diameters of 1 and 2 µm at two concentrations (10^6^ and 10^7^ beads × mL^−1^). The results of the experiments showed that MPs have a variable, species-specific effect on the population growth rate of ciliates, which is directly dependent on their concentration in the environment (*P* < 0.01). It was also observed that the number of MPs ingested changed over time depending on their concentration and size. On average, the highest number of ingested MPs (883.11 ± 521.47) was recorded at 60 min of exposure to a low concentration of small beads in *B. japonicum*. The lowest number of beads was ingested after 5 min of exposure to a low concentration of large beads in the same species. The rate of MP uptake by the ciliate species was significantly dependent on their concentration, exposure time, and size (*P* < 0.001). The highest clearance rate was observed in the fifth minute of the experiment in the environment with the lowest MP concentration.

## Introduction

The history of plastic pollution research within the scientific community began in 1972 when the abundance of small floating particles on the surface of the Atlantic Ocean was first described (Carpenter and Smith 1972; Carpenter et al. 1972). The majority of published studies and reports on plastic pollution have focused on relatively large debris that is hazardous to marine mammals, birds, or fish (Derraik 2002; Amélineau et al. 2016). However, over the past few decades, a large body of research has focused on smaller, less visible plastic debris, known as microplastics (MPs) (Weis 2020; Issac and Kandasubramanian 2021). By definition, MPs are a diverse mixture of differently structured materials. Depending on their shape, MPs can be classified as fibres, spheroids, granules, flakes, beads, or irregularly structured particles. They range in diameter from 0.1 to 5.0 mm and can be classified as primary or secondary microplastics, with the latter being formed by the fragmentation of larger objects (EFSA 2016). Microplastics enter the environment in many ways, but the most common is through the washing of synthetic clothes and abrasion of car tyres. In addition to shopping bags and plastic fishing nets, MPs can also be found in various cosmetics, such as scrubs, hair shampoos, and facial cleansers (Boucher and Friot 2017; Guerranti et al. 2019). The complexity and diversity of MPs are immense. They originate from a variety of sources and product types composed of different polymers, which affects their morphology, and show a wide range of sizes and shapes and incorporation of different mixtures of chemical additives (Thompson et al. 2009). Given their heterogeneity, Rochman et al. (2019) argued that we should not refer to MPs as a single pollutant but rather as a diverse set of pollutants, as is done in the case of pesticides or heavy metals. The term plastics is used to describe a wide range of materials, comprising some 20 different groups, each with many grades and sub-groups (APME 2006). Plastics are incredibly versatile materials given their great adaptability, corrosion resistance, and high thermal and electrical insulation properties. They are affordable, chemical- and water-resistant, strong, durable, and lightweight (Andrady and Neal 2009; Plastics Europe 2010). Moreover, the same properties that make plastics so extraordinary have become a major environmental concern when they end up as waste (Cole et al. 2011). Plastics are an overused material due to their durability, but at the same time, their remarkably long decomposition time and resistance to degradation make them problematic to dispose of (Barnes et al. 2009; Sivan 2011; Cole et al. 2011). In addition, as marine and freshwater contaminants, MPs are widely dispersed in aquatic environments due to their buoyancy and durability properties (Lusher 2015). As observed by Lobelle and Cunliffe (2011), these physicochemical properties of MPs can change rapidly due to the development of microbial films on the surface of submerged particles, which can eventually lead to the sinking of fragments and their accumulation on the bottom of water bodies (Barnes et al. 2009). In addition, waterborne pollutants with hydrophobic properties, most of which are toxic and persistent in the environment, can be adsorbed onto plastic waste, which can enhance their persistence, and be transported with them (Teuten et al. 2009; Thompson et al. 2009; Cole et al. 2011; Engler 2012; Fries et al. 2013; Velzeboer et al. 2014). Although MPs pose a number of threats to aquatic ecosystems, the greatest hazard is their small size, which allows them to enter the aquatic food web at very low trophic levels and diffuse to higher levels or even to terrestrial organisms (Wright et al. 2013; Besseling et al. 2014), as small plastic fragments are easier to assimilate into biological processes due to their favourable surface-to-volume ratio (Zarfl and Matthies 2010; Jeong et al. 2016). The ingestion and accumulation of MPs by many different organisms leads to their accumulation in the food chain, with particular emphasis on organisms at the highest trophic levels (Vandermeersch et al. 2015). Microplastics are available for consumption by a wide range of different aquatic organisms due to their small size, which overlaps with the size range of typical prey (Galloway et al. 2017). They have been found in the guts of zooplankton, invertebrates, molluscs, fish, and other larger animals, including those intended for human consumption and those that play important ecological roles (Browne et al. 2008; Lusher 2015; Galloway et al. 2017; Stienbarger et al. 2021). In addition, ingestion of MPs can have serious consequences, such as clogging of the digestive tract, resulting in impaired food intake and digestion (Cole et al. 2013; Setälä et al. 2014; Canniff and Hoang 2018). Furthermore, most laboratory experiments confirm the adverse effects of MPs on the consumption, nutrition, fertility, growth, development, and lifespan of the organisms tested (Lee et al. 2013; Cole et al. 2013, 2015). However, information on the consumption of MPs and their potential accumulation in naturally occurring populations is still lacking (Lusher et al. 2013).

Diverse groups of zooplankton exhibit a variety of feeding strategies, including consumption of suspended organic matter, filter feeding, detritivory, and predation, which can vary with species, life stage, and prey availability (Strickler 1982; Wirtz 2012). The ingestion of MPs by pelagic filter-feeding zooplankton is well documented, but freshwater ciliates are a prominent group of zooplankton that has been understudied (Bulannga and Schmidt 2022). Heterotrophic protists play an integral role in the aquatic ecosystem in the microbial cycle, as they feed on bacteria and other types of microorganisms such as algae, other protists, and even rotifers (Foissner et al. 1991, 1992, 1999). Microplastic particles, measuring approximately 1–2 µm in size, can be ingested unintentionally or accidentally by ciliates and transferred to higher trophic levels, when protists are ingested by crustacean zooplankton and other small organisms, thus linking all parts of the food web (Cole et al. 2013). However, some filter-, suspension-, and detritus-feeding ciliates living in water and sediments have been observed to intentionally ingest MPs in laboratory experiments (Nugroho and Fyda 2020; Nałęcz-Jawecki et al. 2021; Bulannga and Schmidt 2022). Some ciliates that exhibit the above consumption modes may feed on bacteria, suspended organic matter, or small algae but may also graze on fine inorganic particles (Bernard and Rassoulzadegan 1990; Pierce and Turner 1992). It is therefore of great importance to investigate the ability to ingest plastic microparticles and its effects among zooplankton, including protozoa, which are essential to the aquatic food web.

The primary aim of this study was to describe the effects of MP ingestion on the viability of freshwater ciliates. A secondary aim was to determine the ability of these organisms to ingest MPs and whether there are differences in the number of particles ingested among species at different times of exposure, MP sizes, and concentrations. The specific research objectives were (1) to determine whether the population growth rate is affected by microplastics, (2) to calculate the number of ingested microbeads, and (3) to evaluate the clearance rate in different experimental settings.

## Materials and methods

### Culture and maintenance of ciliated protozoa

The ciliated protozoa used in this study were obtained from permanent cultures maintained at the Aquatic Ecosystems Laboratory at the Institute of Environmental Sciences Jagiellonian University, Poland. From these stock cultures, several clones of *Blepharisma japonicum* Suzuki, 1954, *Spirostomum teres* Claparède and Lachmann, 1858, and *Euplotes* sp. were established using a Zeiss Stemii 2000 stereomicroscope and fine glass micropipettes in 24 well tissue plates (TPP, Switzerland) filled with 500 µL of spring water (Żywiec Zdrój, Poland) as medium. A drop of unidentified bacteria growing on the same medium and wheat grain for *B. japonicum* and *S. teres* and a drop of a dense culture of the flagellate *Chilomonas* sp. for *Euplotes* were added as food. The clones were kept in a SANYO MLR-350 climate chamber at 20 ± 1 °C in the dark. After a few days, the most numerous clones were transferred to Petri dishes of 5 cm in diameter filled with 5 ml of medium. Wheat grains were added as a nutrient source for the bacteria. All clones were kept in the same climate chamber under the same conditions and monitored twice a week. Once a high number of individuals had been reached, at 24 h before the start of the experiment, the ciliates were transferred to clean medium to remove any residual culture. Individuals belonging to the same clone were then randomly selected for each experiment.

### Microplastic particles

Spherical, carboxylate-modified polystyrene latex beads with yellow-green fluorescence of 2 µm (large) and 1 µm (small) diameter, supplied by Sigma-Aldrich (Poland), and stored in the dark at 4 °C were used in the experiments. The maximum excitation wavelength of the microbeads was λ_ex_ ~ 441 nm, and the maximum emission was λ_em_ ~ 486 nm, which is a fluorescence spectrum similar to that of fluorescein isothiocyanate (FITC) (λ_ex_ ~ 491 nm, λ_em_ ~ 516 nm). For experiments, 10 µL of microbead suspension was transferred to 240 µL of deionised water to dilute the original concentration of microbeads. To determine the exact concentration of microbeads in a well, the beads were counted in a Bürker chamber, and their concentration was determined. The low concentration was obtained by pipetting 1 µL of small-bead suspension or 3 µL of large-bead suspension. The high concentration was obtained by pipetting 5 µL of small-bead suspension and 35 µL of large-bead suspension into a well filled with 400 µL of water. The final concentrations were 10^6^ and 10^7^ beads per mL for the low and high concentrations, respectively.

### Influence of MPs on the population growth rate

Population growth rates (PGRs) were expressed as the increase in the number of individuals per day of both control and MP-exposed individuals and were measured in 24-well tissue culture plates. Three cells of each ciliate species were placed in 200 µL of Żywiec Zdrój spring water, and food was provided ad libitum. An appropriate amount of microbeads of each size representing the control, low, and high concentrations was then added. Each experimental setup consisted of 3 replicates. Ciliates were counted in the tissue culture plate after 24, 48, and 72 h of incubation. Tissue culture plates containing ciliates were stored in the dark at room temperature during the experiment. Population growth rates were calculated using the equation described by Fyda and Wiackowski (1998) and modified to fit the data obtained: $\mu =\frac{\left({\text{ln}}\left(N+1\right)-{\text{ln}}\left({N}_{0}+1\right)\right) }{x}$, where *N*_0_ = 3, *N* is the final number of cells and *x* is the subsequent day of exposure to MPs.

### Uptake of plastic microbeads

The experiments were performed in 24-well tissue culture plates. Each well was filled with 400 µL of Żywiec Zdrój spring water, to which 50 individuals randomly taken from monoclonal cultures and one of the two concentrations of microsphere sizes were added. Each experiment consisted of 4 replicates. Then, after 5, 30, or 60 min in different setups, the experiment was terminated by adding an equal amount of 4% formaldehyde for *B. japonicum* and *Euplotes* sp. or methylcellulose for *S. teres*. In brief, 7–11 randomly selected protozoa were removed from each well with a glass micropipette, each of which was treated as a replicate for subsequent microscopic analysis. The cells were then placed on a microscope slide and covered with a coverslip. Each slide was briefly observed under a bright field microscope to identify individuals for subsequent analysis.

Samples were examined using a Nikon Eclipse E-80 epifluorescence microscope. Photographs of randomly selected individuals of *B. japonicum*, *Euplotes* sp., and *S. teres* for further analysis were taken using a Nikon DS-Ri2 DO camera and the NIH program at 400 × magnification. The images were then used to calculate the amount of microplastic beads ingested by each ciliate species using ImageJ software. To calculate the actual number of microspheres absorbed, it was assumed that the MP particles were round in a 2-dimensional visualization. Calculations were made using a scale where 100 µm at 400 × magnification corresponds to 544 pixels in ImageJ software. ImageJ tools were then used to determine the area occupied by the food vacuole filled with the collected MPs, to calculate the area of a MP sphere, and to calculate the number of spheres ingested by the ciliates by dividing the area of the vacuole by the area of a microsphere.

### Clearance rate

To determine the clearance rate (C) per individual in each experiment at different concentrations and times, the calculation described by Peters (1984) was followed: $C =\frac{B}{\left(S\cdot T\right)} ,$ where *B* = beads counted inside the cell, *S* = concentration, and *T* = time of exposure of the cells to the bead suspension. The clearance rate for individuals was expressed as the number of beads ingested × concentrations^−1^ × time^−1^.

### Statistical analysis

A three-factor factorial model and ANOVA were used to test whether MP concentration, exposure time, and size (fixed factors) influenced the number of ingested microbeads (dependent variable). The interaction between the variables was also taken into account in the model. Separate analyses were performed for each ciliate species.

The effects of MP concentration, size, and exposure time (fixed factors) on clearance rate (dependent variable) were tested using three-way ANOVA. A new model was fitted independently for each species. Each model included an interaction between the variables.

Three-way ANOVA was adapted to test whether the concentration of MPs in the water, their size, and exposure time, considered fixed factors, had an effect on the PGR, which was considered the dependent variable. Discrete models were fitted for each ciliate species, and all models took into account the interaction between concentration, size, and time. Interactions that did not show statistical significance were later removed from the models.

In addition, to test whether the size of the ciliate cell influenced the uptake and clearance rate of MPs, a linear model was fitted with cell area as the independent variable and bead uptake and clearance rate as the dependent variables. Statistical analyses and plotting were performed in R Studio (R Core Team, 2020). A *P* value = 0.05 was considered a significant difference between groups in all tests performed.

## Results

### Influence of MPs on the population growth rate

**Fig. 1:**
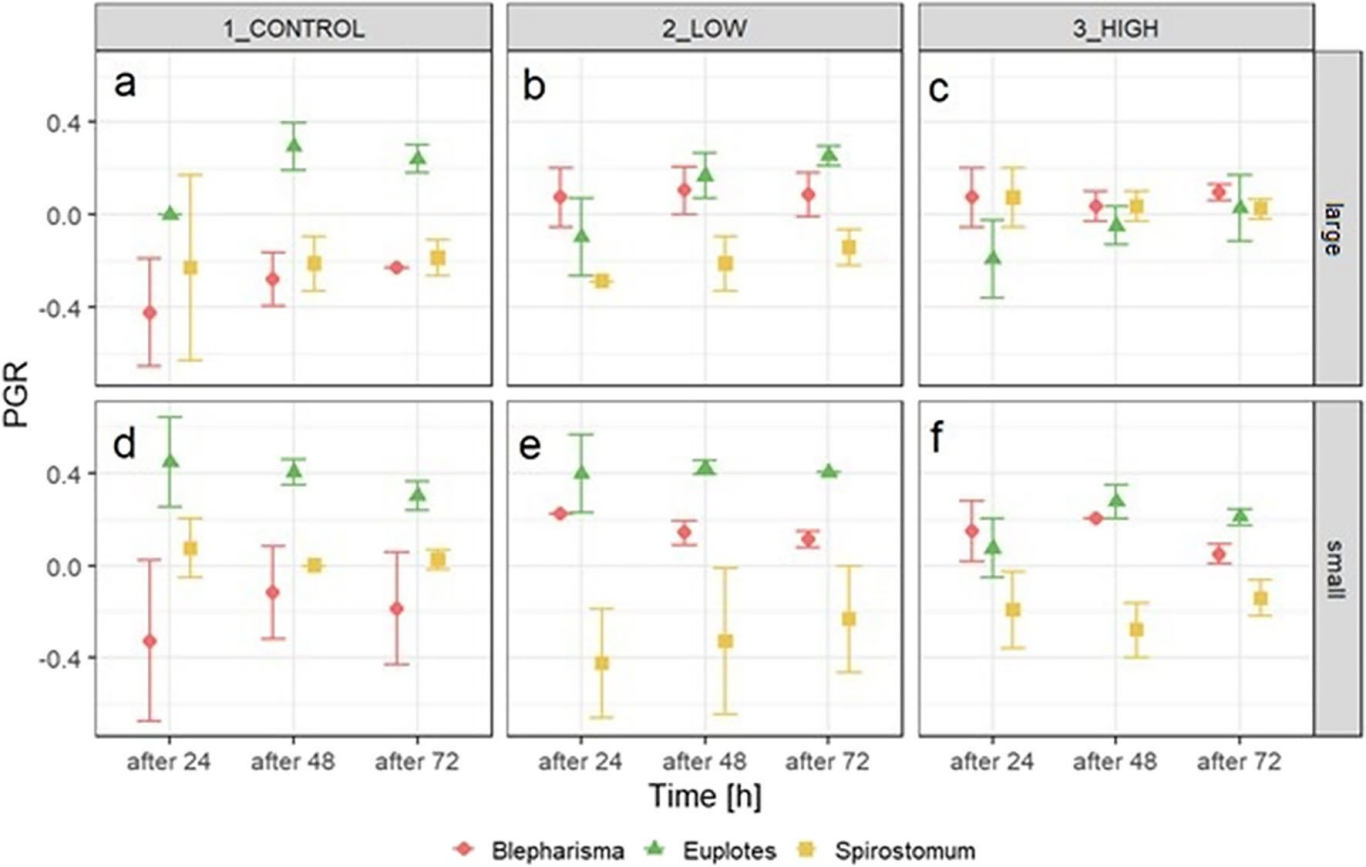
Comparison of mean (± SD) population growth rates (PGRs) of ciliates at different microparticle exposures times (24, 48, 72 h), concentrations (10^6^ vs. 10^7^ beads × mL.^−1^) and sizes (small, 1 µm vs. large, 2 µm)

The PGR varied among species and different experimental conditions. In the control, the population growth rate of *B. japonicum* was observed to be negative on each day of the experiment. A similar result was observed in the experiment with *S. teres* and large particles at low concentrations. In contrast, for *B. japonicum* in the experiment with small particles, the population growth rate was positive. Similar to *Blepharisma*, an even more pronounced result was observed for *Euplotes* sp., with PGR values reaching µ = 0.2–0.4 in the experiment with small plastic particles (Fig. 1e, f).

**Fig. 2:**
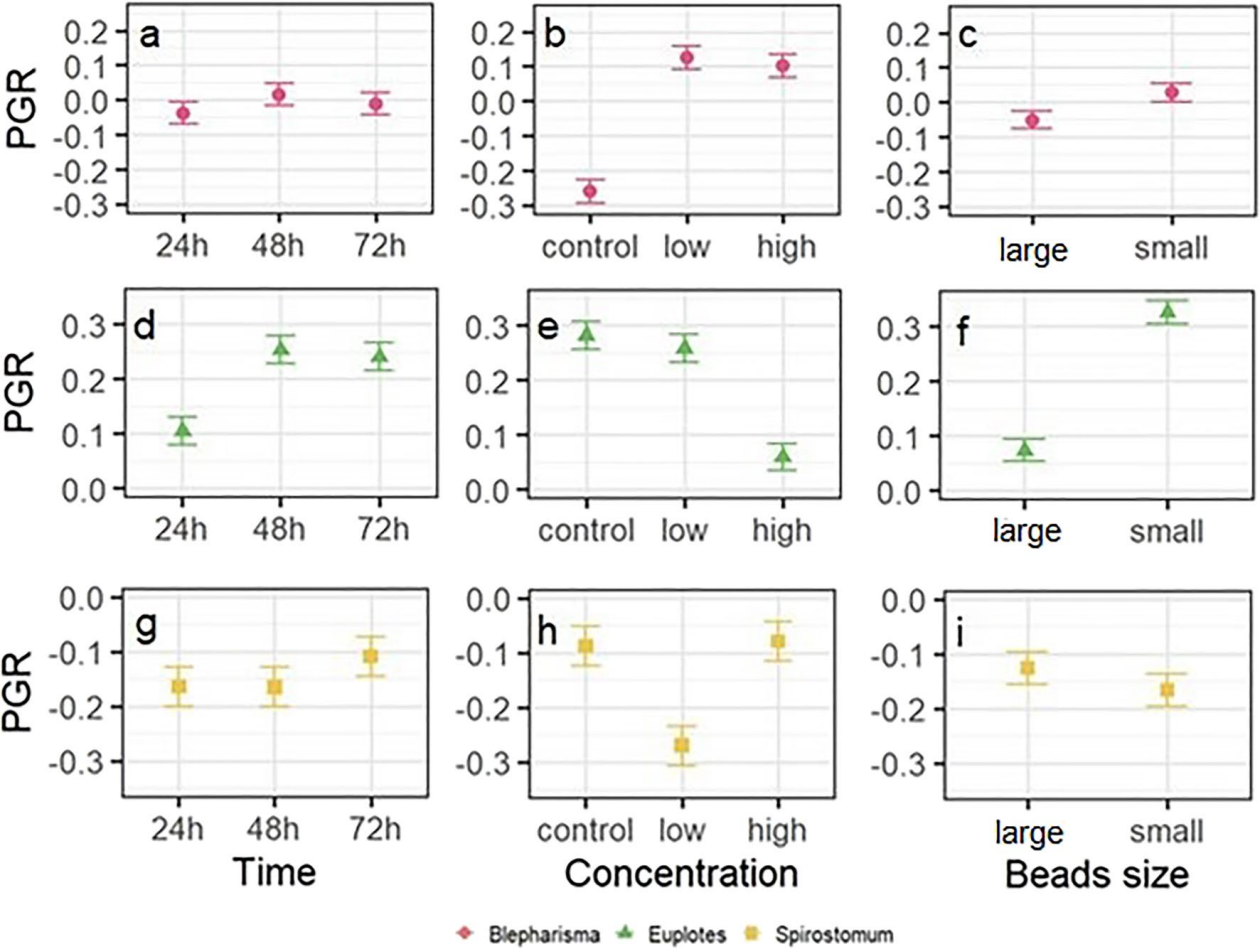
Effect of microparticle concentration, size, and exposure time on the population growth rate (PGR) of *B. japonicum* (**a**–**c**), *Euplotes* sp. (**d**–**f**), and *S. teres* (**g**–**i**). Plots show the least squares (LS) means of PGR (± SEs)

The growth rate of *B. japonicum* was influenced by both the concentration and size of the microbeads (Table 1). A high PGR was observed at low concentration of small microbeads (Fig. 1e). A post hoc test showed that there was no difference between the low and high concentrations, but a highly significant difference (*P* ≤ 0.0001) was observed between the remaining treatments. The effect of size was most pronounced at lower PGR levels in the large bead treatment (Fig. 2c, f).

**Table 1:** Results of the ANOVA comparing the population growth rate of each ciliate species exposed for different times to different microplastic concentrations and sizes

Population growth rate	SS	*df*	MS	*F*	*P*
*Euplotes* sp.					
Time***	0.24	2	0.12	10.629	*P* < 0.001
Concentration***	0.54	2	0.26	23.709	*P* < 0.001
Size**	0.87	1	0.87	76.055	*P* < 0.001
Time/size**	0.17	2	0.08	7.532	*P* < 0.01
Time/concentration	0.04	4	0.01	0.849	*P* > 0.05
Concentration/size	0.02	2	0.01	0.847	*P* > 0.05
Time/concentration/size	0.07	4	0.02	1.577	*P* > 0.05
Residuals	0.41	36	0.01		
*Spirostomum teres*					
Time	0.04	2	0.02	0.069	*P* > 0.05
Concentration**	0.42	2	0.21	7.567	*P* < 0.01
Size	0.02	1	0.02	0.787	*P* > 0.05
Time/size	0.01	2	0.005	0.147	*P* > 0.05
Time/concentration	0.07	4	0.02	0.599	*P* > 0.05
Concentration/size***	0.58	2	0.29	10.521	*P* < 0.001
Time/concentration/size	0.02	4	0.005	0.180	*P* < 0.05
Residuals	0.99	36	0.03		
*Blepharisma japonicum*					
Time	0.03	2	0.01	0.062	*P* > 0.05
Concentration***	1.67	2	0.84	40.793	*P* < 0.001
Size*	0.08	1	0.08	4.130	*P* < 0.05
Time/size	0.03	2	0.01	0.819	*P* > 0.05
Time/concentration	0.11	4	0.03	1.311	*P* > 0.05
Concentration/size***	0.003	2	0.001	0.084	*P* > 0.05
Time/concentration/size	0.02	4	0.006	0.293	*P* > 0.05
Residuals	0.74	36	0.02		

^*^Significant at the 0.05 probability level

^**^Significant at the 0.01 probability level

^***^Significant at the 0.001 probability level

The PGR of *Euplotes* sp. was significantly dependent on exposure time, microbead concentration and size, as well as on the interaction between exposure time and microbead size (Table 1). The results show a difference in the PGR between the treatments with two different MP sizes, with higher PGR values observed after exposure to smaller microbeads. The post hoc Tukey test was performed to assess between which exposure times the PGR differed. The results show a significant difference between 24 and 48 h (*P* = 0.0005) and between 24 and 72 h (*P* = 0.0015). There was no significant difference between 48 and 72 h of exposure. Due to interaction effects, this is only applicable when considering the size of the MPs. In addition, the same post hoc test was performed to determine differences between concentrations. In this case, no difference was found between the control and low concentrations, but significant differences (*P* < 0.0001) were found between the control and high concentrations and between the low concentration and high concentrations (Fig. 2b, e, h).

For *S. teres*, the PGR was significantly dependent on concentration and the interaction between concentration and particle size (Table 1). The post hoc Tukey test showed significant differences between the control and low concentrations (*P* = 0.0026) and between the low and high concentrations (*P* = 0.0015) (Fig. 2h). Because of the interaction involved, it is crucial to consider the effect of concentration in relation to microparticle size. At low concentrations, there seems to be no difference between the PGR observed for both microparticle sizes. Conversely, at high concentrations, the PGR was lower in the treatment with smaller beads (Fig. 2i).

### Plastic microbead uptake

**Fig. 3:**
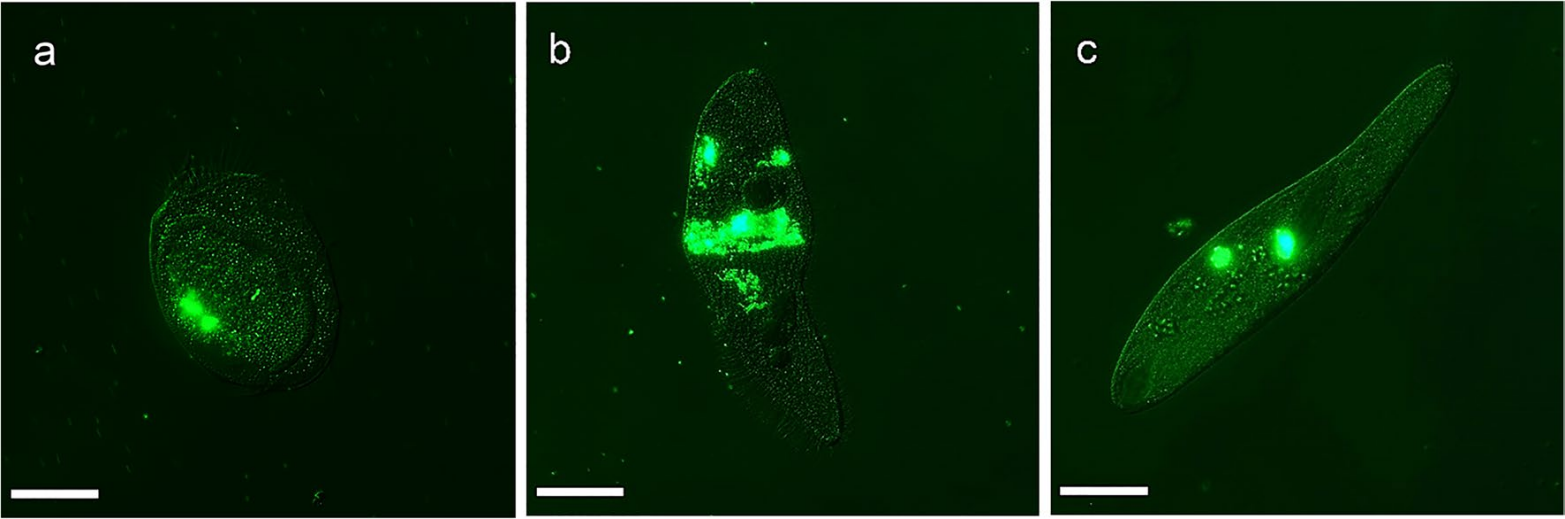
Fluorescently labelled microplastic beads ingested by the ciliates *Euplotes* sp. (**a**), *Blepharisma japonicum* (**b**), and *Spirostomum teres* (**c**), visible under epifluorescence microscopy. The bar represents 50 µm

The results of the experiment showed that ciliates readily ingested microplastic particles (Fig. 3). In the case of *Euplotes* sp. and *S. teres*, both ciliate species showed positive ingestion of microbeads in all experimental setups. In contrast, no beads were found in the food vacuoles of some individuals of *B. japonicum* at low concentrations of large microbeads after 5 min of exposure. The maximum number of ingested particles varied among species. For *Euplotes* sp., the highest number of MPs was achieved at the high concentration at 60-min exposure time with small beads, reaching 280.83 ± 150.42 beads per individual.

**Fig. 4:**
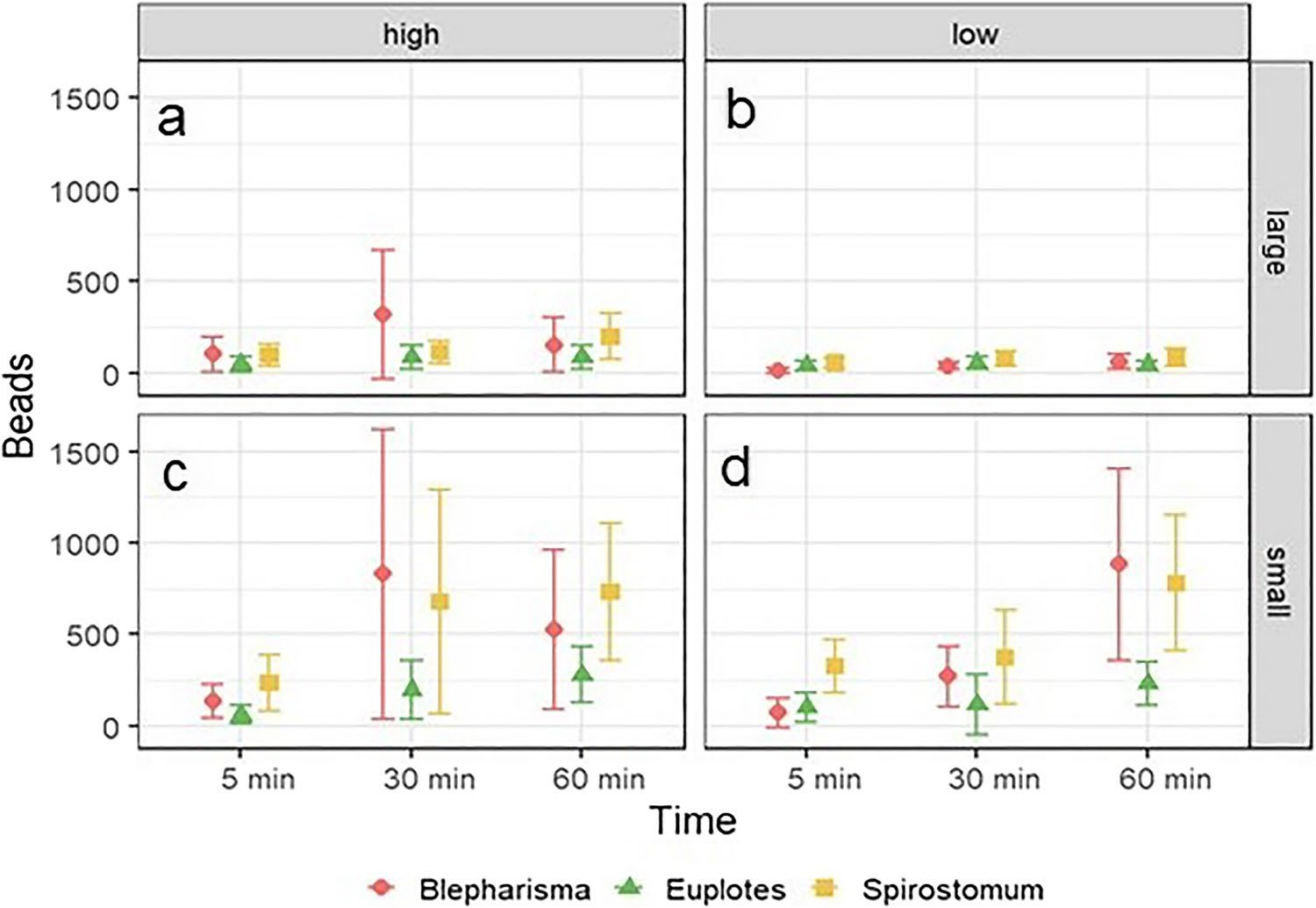
Comparison of the mean number (± SD) of two sizes of microplastic beads ingested by ciliates at different concentrations and exposure times

For *B. japonicum* and *S. teres*, MP uptake peaked at low concentrations of smaller beads at 60 min of exposure time, reaching 779.28 ± 371.10 and 883.11 ± 521.47 beads, respectively (Fig. 4d).

**Fig. 5:**
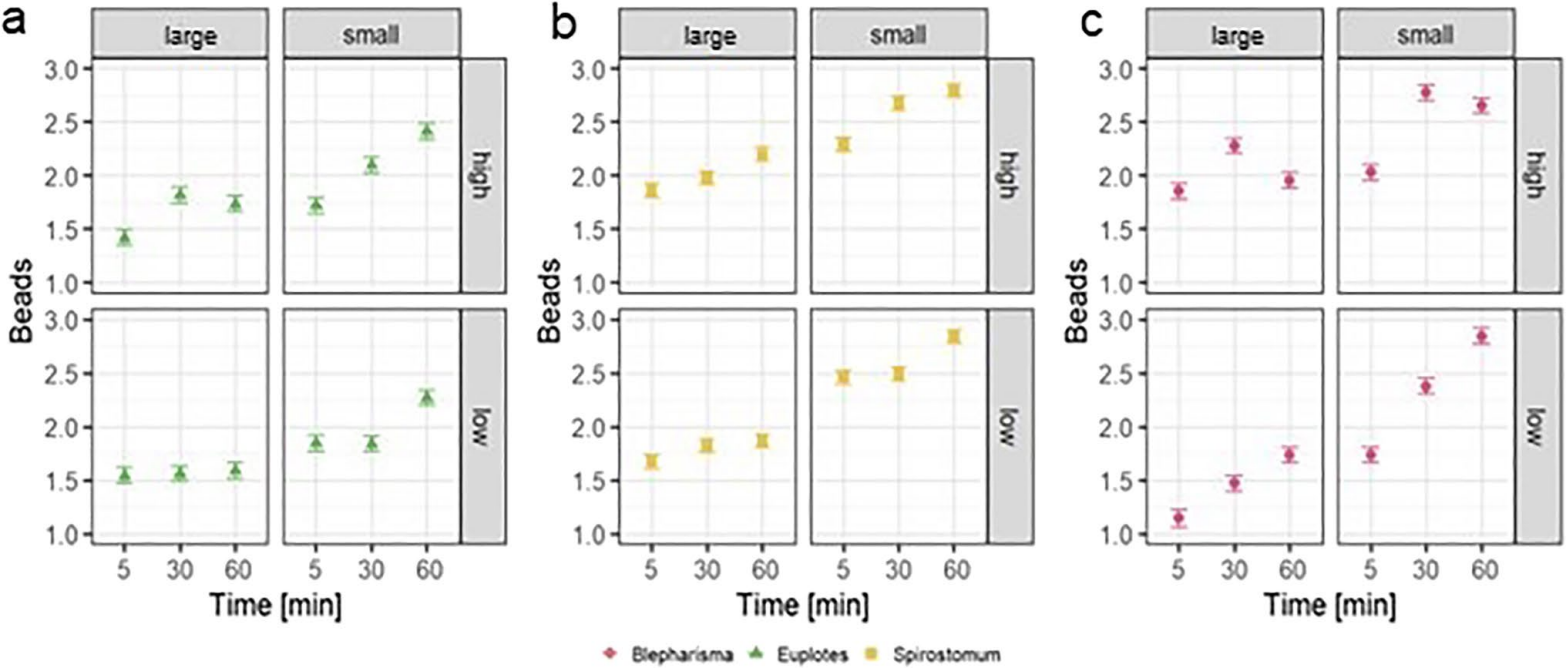
Effect of microparticle concentration, size and exposure time on the least squares (LS) mean number (± SDs) of beads ingested by *Euplotes* sp. (**a**), *Spirostomum teres* (**b**), and *Blepharisma japonicum* (**c**)

In *B. japonicum*, the number of microbeads ingested was significantly influenced by the variables individually as well as by their first-order interactions (Table 2). Grazing was greater for beads of smaller size as well as for beads at high concentrations. For the low concentration, the number of ingested beads increased with exposure time, whereas for the high concentration, the number of ingested MPs peaked at 30 min and decreased thereafter. Moreover, a similar effect can be observed for large MPs, where ingestion peaked at 30 min decrease thereafter. For small beads, uptake increased with increasing exposure time (Fig. 5). Furthermore, the Tukey test showed that ingestion at the 5-min exposure time was significantly different from ingestion at 30 and 60 min (*P* < 0.0001).

**Table 2:** Results of the ANOVA comparing the number of microbeads ingested by each ciliate species exposed to different MP concentrations and sizes for different lengths of time

Number of ingested beads	SS	*df*	MS	*F*	*P*
*Euplotes* sp.					
Time***	5.48	2	2.74	19.138	*P* < 0.001
Concentration	0.48	1	0.48	3.365	*P* > 0.05
Size***	10.55	1	10.55	73.590	*P* < 0.001
Time/size**	1.99	2	0.99	6.933	*P* < 0.01
Time/concentration**	1.53	2	0.77	5.349	*P* < 0.01
Concentration/size	0.01	1	0.01	0.06	*P* > 0.05
Time/concentration/size	0.16	2	0.08	0.545	*P* > 0.05
Residuals	32.68	228	0.14		
*Spirostomum teres*					
Time***	5.03	2	2.51	31.253	*P* < 0.001
Concentration**	0.66	1	0.66	8.192	*P* < 0.01
Size**	28.67	1	28.67	356.373	*P* < 0.001
Time/size	0.29	2	0.15	1.861	*P* > 0.05
Time/concentration	0.29	2	0.15	1.829	*P* > 0.05
Concentration/size**	0.84	1	0.84	10.486	*P* < 0.01
Time/concentration/size*	0.52	2	0.26	3.241	*P* < 0.05
Residuals	18.35	228	0.08		
*Blepharisma japonicum*					
Time***	14.26	2	7.13	58.645	*P* < 0.001
Concentration***	7.15	1	7.15	58.836	*P* < 0.001
Size***	25.49	1	25.49	209.791	*P* < 0.001
Time/size***	2.83	2	1.42	11.659	*P* < 0.001
Time/concentration***	3.95	2	1.97	16.235	*P* < 0.001
Concentration/size***	2.49	1	2.49	20.536	*P* < 0.001
Time/concentration/size	0.29	2	0.15	1.219	*P* > 0.05
Residuals	26.98	222	0.12		

^*^Significant at the 0.05 probability level

^**^Significant at the 0.01 probability level

^***^Significant at the 0.001 probability level

The uptake of MPs by *Euplotes* sp. was mainly determined by exposure time, sphere size, and their interaction. The effect of concentration was only significant in the interaction with exposure time (Table 2). The number of beads ingested was generally greater for small beads than for large beads at each concentration. In the experiment with smaller microbeads, the number of MPs ingested increased with exposure time, but for exposure to large microbeads, ingestion did not increase with longer times. A similar result is observed for the effect of concentration, with the number of ingested beads increasing with longer exposure times (Fig. 5). In addition, a post hoc Tukey test was performed to test which exposure times differed from each other. The results show that the number of ingested MPs differed between all exposure times.

The grazing of *S. teres* on MPs was dependent on time, concentration and size of MPs, the interaction of concentration and size of MPs and most importantly the interaction of all three factors (Table 2). The results show an increasing number of microspheres ingested with increasing exposure time, with the highest number of beads ingested at 60 min. In addition, a post hoc test showed that there was a significant difference in the number of MPs ingested at each exposure time. Finally, small beads were preferred by this ciliate and ingested in greater numbers than large beads (Fig. 5b).

### Clearance rate

**Fig. 6:**
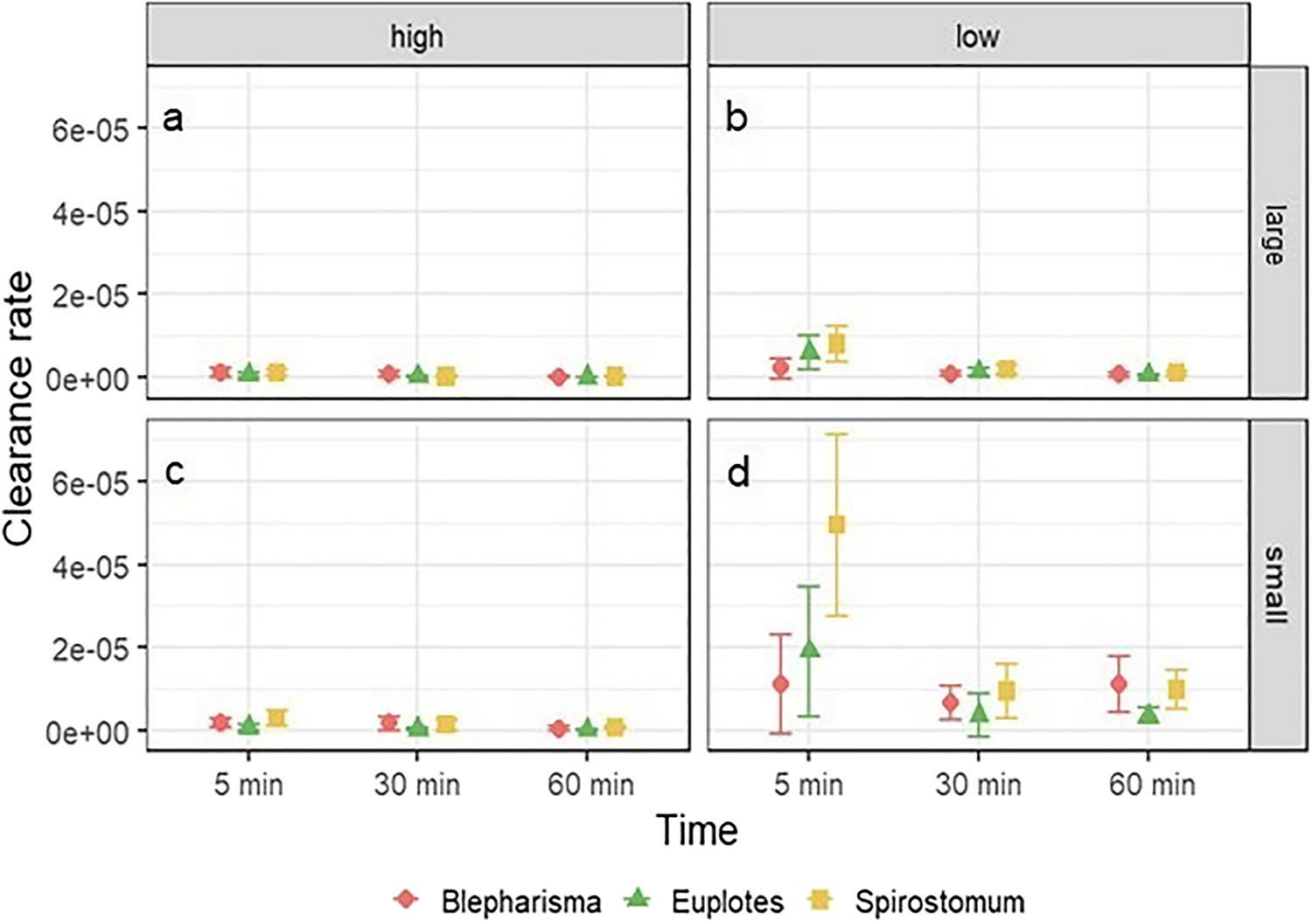
Comparison of the mean clearance rate (± SD) of ciliates at different exposure times, concentrations and microparticle sizes

Microplastics were cleared at the highest rate by *S. teres* at low concentrations at 5 min of exposure time for small and large particles, reaching 4.95 × 10^−5^ and 8.06 × 10^−6^, respectively (Fig. 6b, d). *Euplotes* sp. achieved the second highest clearance rate at 5 min of exposure at low concentrations, reaching 1.92 × 10^−5^ for small beads and 6.1 × 10^−6^ for large beads. The lowest clearance rate was observed for *B. japonicum* under the same conditions and reached 1.12 × 10^−5^ for small beads and 2.1 × 10^−6^ for large beads (Fig. 6b, d).

In *B. japonicum*, the clearance rate was significantly dependent on all three tested factors as well as their first-order interactions (Table 3). At the 5th minute of exposure, the clearance rate was highest for both concentrations. For the high concentration, the clearance rate gradually decreased with exposure time, whereas for the low concentration, the clearance rate was lowest in the 30th min and then increased in the 60th min. The post hoc test showed that the clearance rate at each exposure time was significantly different from each other (*P* = 0.0001). For large beads, the clearance rate was higher than for small beads (Fig. 6).

**Table 3:** Results of the ANOVA comparing the clearance rates of each ciliate species exposed for different times to different concentrations and sizes of MPs

Clearance rate	SS	*df*	MS	*F*	*P*
*Euplotes* sp.					
Time***	22.93	2	11.47	80.138	*P* < 0.001
Concentration***	64.70	1	64.70	452.151	*P* < 0.001
Size***	13.09	1	13.09	91.481	*P* < 0.001
Time/size**	1.97	2	0.99	6.893	*P* < 0.01
Time/concentration**	1.53	2	0.77	5.373	*P* < 0.01
Concentration/size	0.07	1	0.07	0.516	*P* > 0.05
Time/concentration/size	0.16	2	0.08	0.541	*P* > 0.05
Residuals	32.63	228	0.14		
*Spirostomum teres*					
Time***	24.17	2	12.08	150.171	*P* < 0.001
Concentration***	57.15	1	57.15	710.333	*P* < 0.001
Size***	28.67	1	28.67	356.373	*P* < 0.001
Time/size	0.29	2	0.15	1.861	*P* > 0.05
Time/concentration	0.29	2	0.15	1.829	*P* > 0.05
Concentration/size**	0.84	1	0.84	10.486	*P* < 0.01
Time/concentration/size*	0.52	2	0.26	3.241	*P* < 0.05
Residuals	18.35	228	0.08		
*Blepharisma japonicum*					
Time***	8.57	2	4.29	32.264	*P* < 0.001
Concentration***	31.18	1	31.18	256.523	*P* < 0.001
Size***	25.49	1	25.49	209.795	*P* < 0.001
Time/size***	2.83	2	1.42	11.659	*P* < 0.001
Time/concentration***	3.95	2	1.97	16.235	*P* < 0.001
Concentration/size***	2.49	1	2.49	20.537	*P* < 0.001
Time/concentration/size	0.29	2	0.15	1.219	*P* > 0.05
Residuals	26.98	222	0.12		

^*^Significant at the 0.05 probability level

^**^Significant at the 0.01 probability level

^***^Significant at the 0.001 probability level

The clearance rate of *Euplotes* sp. was significantly influenced by exposure time, concentration, and size of the MPs. In addition, the interaction between time and concentration and time and particle size was also significant (Table 3). At lower particle concentrations, the clearance rate was highest at the 5th minute of exposure and then decreased at the 30th and 60th min. The same effect was observed at the high concentration. Post hoc tests showed no differences between the 30th and 60th min of exposure (*P* = 0.08). For small microbeads, the clearance rate decreased from the 5th to the 30th min and then increased at the 60th min. However, for large particles, the clearance rate decreased progressively (Fig. 6).

**Fig. 7:**
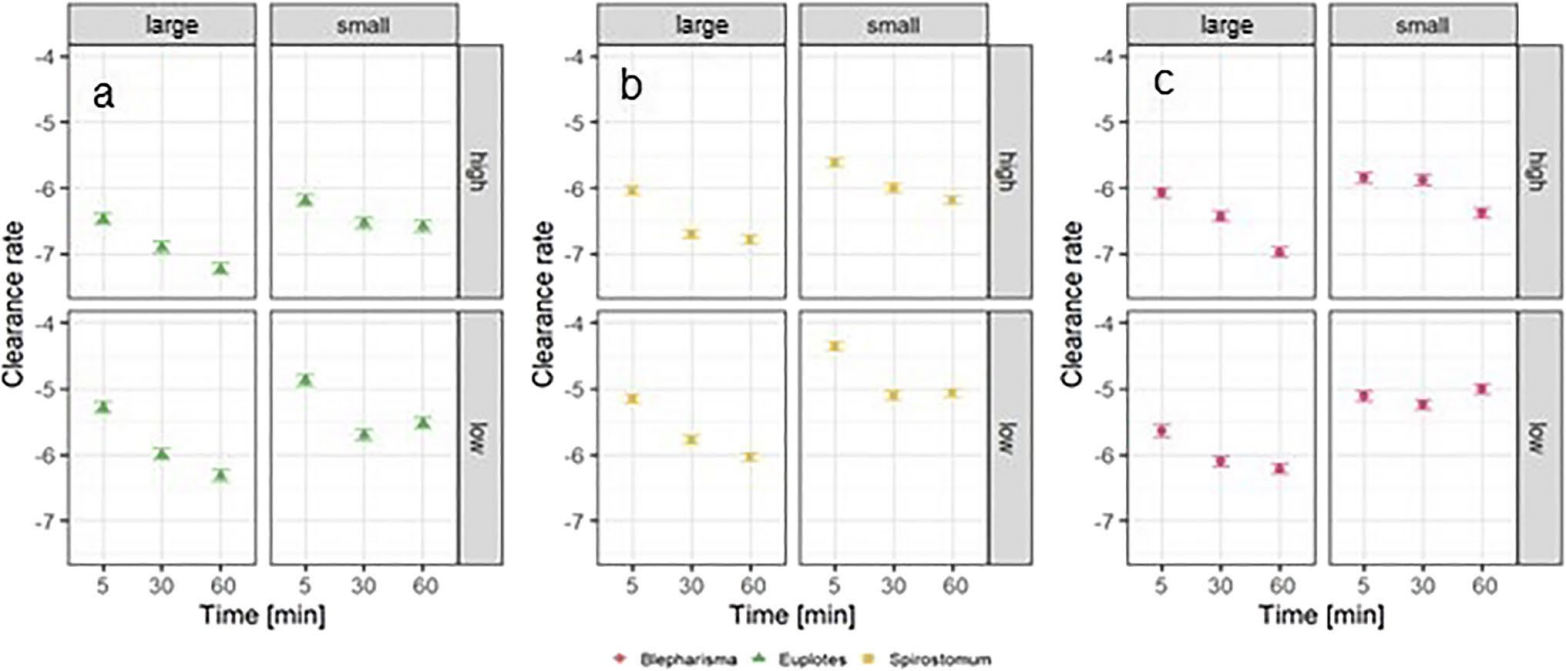
Effect of microparticle concentration, size, and exposure time on the least squares (LS) mean (± SDs) clearance rate of *Euplotes* sp. (**a**), *Spirostomum teres* (**b**), and *Blepharisma japonicum* (**c**)

The clearance rate of *S. teres* was determined by the concentration and size of the microparticles as well as by the exposure time. In addition, the interaction of concentration and size as well as the second level interaction of time, concentration and size were also significant (Table 3). The clearance rate observed in the experiment with small beads was higher than that observed in the experiment with large beads in this species. A similar result was observed for concentration, with a higher clearance rate at high than low concentration. Finally, in each experimental condition, the clearance rate decreased with increasing exposure time, although the post hoc test showed no significant differences between the 30th and 60th minute of exposure (Fig. 7a–c).

**Fig. 8:**
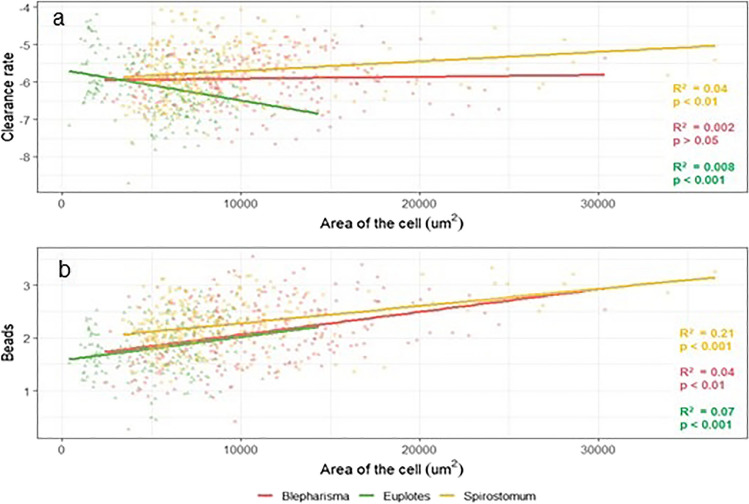
Correlation between clearance rates (**a**) and the number of ingested beads (**b**) and cell area of each ciliate species. The plots show the least squares (LS) mean number of ingested beads and clearance rate (± SEs) a logarithmic scale

The results show that the area of the ciliate cell correlates with the clearance rate for both *Euplotes* sp. and *S. teres* (Fig. 8a). In *S. teres*, larger organisms were found to graze at a higher rate than smaller individuals. In the case of *Euplotes* sp., the results show the opposite direction to *S. teres*, with smaller cells grazing more effectively. For *B. japonicum*, no significant relationship was found between grazing rate and cell area. Cell area was also significantly positively correlated with the number of beads ingested by each ciliate species (Fig. 8b).

## Discussion

### Population growth rate

This study was designed to determine the effect of MPs on the population growth rate of three ciliates: *Blepharisma japonicum*, *Spirostomum teres*, and *Euplotes* sp. For each experiment, two different sizes (1 µm and 2 µm) and concentrations (10^6^ and 10^7^ beads per mL) of MPs as well as three different time points were used to obtain a thorough picture of the influence of these factors on the viability of the ciliates studied. To date, the literature on the effects of MPs on ciliates remains scarce, and very limited sources are available on the topic of ciliate population growth rates.

To the best of the authors’ knowledge, no studies have investigated the growth rate of *B. japonicum* populations in response to MPs. The results showed that for *B. japonicum*, both MP size and concentration had a significant effect on the growth rate. Interestingly, the population growth rates in the experiment with small beads reached higher values (0.05) than in the experiment with larger beads, in which the population growth rate was negative and measured approximately − 0.05. The second major finding of the experiment was that the population growth rate in the control experiment was negative and much lower than that in the experiments with added microbeads at both concentrations. Although there is no literature on *B. japonicum*, a similar hormesis-like effect can be observed in different species. As observed by Chen et al. (2021), MPs had a positive effect on the growth of marine microalgae when their density was high and concentration per cell was lower. In addition, in a review of the hormetic effects of MPs, Sun et al. (2021) described the positive influence of MPs on the consumption, growth, reproduction and survival of aquatic organisms. It may seem intuitive that MPs, which have no nutritional value and can potentially cause blockage of the digestive tract and impaired feeding, would be harmful to ciliates, but as Paracelsus stated “the dose makes the poison,” and as highlighted in a review by Agathokleous et al. (2021), there may be no adverse effects on organisms below the toxicological threshold, as can be observed in *B. japonicum*.

Despite the interest in the effects of MPs on the genus *Spirostomum*, no studies have considered the effects of MPs on the growth rate of *S. teres*. As already mentioned in the results, the growth rate of the *S. teres* population was dependent on the concentration and its relationship with the size of the microparticles. The same population growth rate was observed for the control and high concentration of MPs, suggesting that there is no visible effect of exposure to a high concentration of MPs. These results are consistent with those of Chojnacka et al. (2023), who studied the joint effects of MPs and antidepressants on the ciliate *Spirostomum ambiguum* and found that MPs in the environment are unlikely to pose a threat to protozoa. Furthermore, Davarpanah and Guilhermino (2015) measured the specific mean growth rate of the green alga *Tetraselmis chuii* and found no significant effects of MPs. In contrast, in the present study, the population growth rate was significantly lower at low concentrations of beads compared to both the control and high concentrations, suggesting a negative effect of MPs on the viability of these species, in contrast to the previous findings of Nałęcz-Jawecki et al. (2021), who described a possible negative effect of nanoparticles on vacuole formation in *S. ambiguum* only when the toxicity threshold was exceeded. These conflicting results may be due to differences in particle concentrations, type of MPs or different ciliate species used in the two studies.

Several studies have found that MPs are harmful to aquatic organisms (Lee et al. 2013; Cole et al. 2013, 2015; Setälä et al. 2014; Canniff and Hoang 2018). In a study of the small planktonic crustacean *Daphnia magna*, the authors found that long-term exposure to MPs had negative effects on their reproduction and population growth rates, especially at elevated water temperature and light intensity (Guilhermino et al. 2021). These results are consistent with our findings that the population growth rate of *Euplotes* sp. decreased from the third day of the experiment and with increasing concentrations of MPs, although it should be noted that the experiments were conducted on different time scales. Furthermore, in a study on *Euplotes vannus*, the consumption of MPs significantly reduced the richness and carbon biomass of the populations (Wang et al. 2022). Furthermore, data presented by the same authors show different patterns in the detrimental effects of different microparticle sizes, with smaller particles being more detrimental, while our results indicate that larger particles had more severe effects on the population growth rate of *Euplotes* sp. Finally, in a study by Shore et al. (2021), MPs were found to reduce the body length and survival of the marine copepod *Acartia tonsa*, with estimates of over 15% reduction in population growth.

### Plastic microbead uptake

The secondary aim of this study was to determine the maximum ingestion capacity of MPs by the three ciliate species studied. As in the previous experiment, two different sizes and concentrations of MPs and three different time points were used to determine the detailed differences in the ingestion of MPs by these species. Previous work in this area has focused primarily on the ingestion of natural foods, and it has not yet been established how many microspheres can be ingested by the species studied in this experiment.

Of all the possible interactions between MPs and aquatic organisms, ingestion is one of the most likely. The microscopic size range of MPs is the reason why they could be ingested by a wide range of biota in aquatic ecosystems. Sometimes organisms are unable to discriminate between natural prey and artificial food (Moore et al. 2001). Furthermore, when MPs are present at high concentrations compared to natural food, organisms may graze on them unintentionally and ingest them accidentally or simply fail to prevent ingestion (Moore 2008; Lusher 2015). During feeding in filtering ciliates, food particles are concentrated by the filtering apparatus and then digested in a food vacuole. In this study, it was observed that ingested MPs were not digested by ciliates and were expelled in the form of compacted masses after approximately one hour. The egestion of MPs in the form of packets filled with smaller particles has also been observed in previous studies (Mueller et al. 1965; Cole et al. 2013). The concentration of food in the environment affects both of the above processes, since both require unrestricted access to food. In filtering ciliates, the concentration of food is the limiting factor if the filtering apparatus cannot collect particles as fast as food vacuoles can be formed. However, if food is ingested at a faster rate than food vacuole formation and lysosomal digestion can handle it, then concentration can be considered a non-limiting factor (Zubkov and Sleigh 1995). In *Euplotes* sp., the number of microspheres ingested depended on the time of exposure and the size of the MPs, as well as the interactions of time with size and concentration. The ingestion of MPs increased with time in almost all experimental setups, except for exposure to low concentrations of large beads, where the highest number was reached at 30 min. In addition, in the *B. japonicum* experiment, the number of ingested beads increased with time only at the low concentration, whereas at the high concentration, the number of ingested beads peaked at 30 min for both MP sizes. Similar results, where equilibrium is reached after some exposure time and then the number of ingested particles decreases, have been obtained in experiments with other ciliate species (Nugroho and Fyda 2020). A reasonable explanation for this result is that, as explained by Zubkov and Sleigh (1995), particle concentration was not a limiting factor. However, the number of beads ingested by *S. teres* increased with increasing exposure time for both concentrations and sizes of MPs. Most likely, these results could be explained by a limit to the capacity of the ciliate food vacuole that must not be exceeded, as in the case of *B. japonicum* and *Euplotes* sp. However, in the case of *S. teres*, the spheres did not reach a critical point as ingestion continued to increase.

All three ciliate species tested are bacterivorous but can also feed on flagellates, algae and other ciliates (Foissner et al. (1991, 1992, 1999). The diet of *B. japonicum* includes a variety of smaller organisms among them bacteria, algae, rotifers, and other ciliates, even individuals of the same species, so prey size varies from 0.5 to 139 µm (Giese 1938). On the other hand, *Spirostomum teres* ingests particles ranging from approximately 0.5 to 100 µm (Finlay and Esteban 1998; Fernandes and da Silva-Neto 2013; Boscaro et al. 2014). *Euplotes* generally feeds on prey from 0.5 to 2 µm but is capable of ingesting larger particles up to 10 µm (Wilks and Sleigh 1998, 2008). For filtering organisms, a clear relationship was found between their morphology and particle size. The minimum particle size is mainly determined by the shape and size of the filtering apparatus. The maximum size of ingested particles is determined by the morphology of the mouthparts (Scherer et al. 2018). Therefore, differences in ingestion may be due to differences in the mechanical properties of the ciliate cytostome, which captures suitable prey and differs in structure in the studied species.

### Particle size preference

Another interesting aspect that emerged from the analysis is that the number of particles ingested is significantly related to both the size of the ciliate and the size of the MPs, with smaller beads being ingested in much greater quantities than the larger beads used in this experiment. Despite the fact that ciliates in general show food selectivity under natural conditions, the sizes of plastic microbeads used in the experiment overlap with the usual prey sizes of each ciliate. Furthermore, previous studies have not addressed particle size preference in *B. japonicum* and *S. teres*, but we have obtained satisfactory results showing that particles of 1 µm in diameter are preferred over particles of 2 µm in both species. The same results were obtained in the experiment with *Euplotes* sp. Our data showed a different trend from that observed by Wilks and Sleigh (1998), who studied the ingestion of microspheres as a function of their size and concentration in the ciliate *Euplotes mutabilis*. In their work, the preference was shifted towards larger particles and confirmed the results of Gonzalez et al. (1990), who showed the preference of ciliates for larger bacteria.

### Clearance rate

During the experiment, we measured the clearance rates of the three investigated species at two different concentrations, three time points, and two particle sizes. The analysis confirmed that the clearance rate was significantly dependent on the factors studied and their interactions. Børsheim (1984) used monodisperse fluorescent latex particles with diameters of 0.57 and 1.04 µm to measure the clearance rates of bacteria-sized particles by two ciliates from a Norwegian lake. The clearance rates of these particles ranged from 0.23 to 1.26 µL ind^−1^ h^−1^ in the case of *Epistylis rotans* and from 0.26 to 0.90 µL ind^−1^ h^−1^ for *Strombidium* sp. Clearance rates varied with food content and temperature.

Our results show that for each species, the clearance rate was highest at the lowest concentration and shortest exposure time. The clearance rate generally decreased with increasing exposure time for both concentrations but was higher for smaller beads for all species. However, for *B. japonicum* and *Euplotes* sp. in the experiment with the low concentration of small beads, the clearance rate was lowest at the 30th minute of incubation. To date, research and reports confirm that the consumption rate of natural food is consistent with estimates of the consumption rate of synthetic particles and that only a limited preference for real cells can be observed compared to inert microspheres (Mueller et al. 1965; Fenchel 1980; Wilks and Sleigh 2008; Weisse et al. 2021).

These results are consistent with those of Peters (1984), who found that the clearance rate of planktonic organisms gradually decreased after peaking, even though food concentrations increased. Similar conclusions were drawn by Nugroho and Fyda (2020) in their experiment with the ciliate *Paramecium aurelia*. Furthermore, our results support Fenchel’s (1980) hypothesis that ciliates have different food preferences that may affect particle ingestion and that their clearance rate decreases as they ingest larger particles. However, for the species used in this experiment, the 1- and 2-µm-diameter microbeads used are an optimal size. Slightly different results were reported by Wilks and Sleigh (2008), who observed that smaller particles, approximately 1 µm in diameter, were ingested in large numbers but less efficiently than larger particles.

However, patterns of MP ingestion by filter-feeding ciliates should not be generalised, as laboratory conditions differ significantly from natural ones, where MPs are not perfectly spherical, absorb chemicals from the environment, harbour bacterial biofilms and can aggregate with each other. For example, Pajdak-Stós et al. (2023) showed that the rotifer *Lecane inermis* preferentially ingests microplastics embedded in biofilm, and that MP aggregation is significantly enhanced by the presence of biofilm, and further enhanced in the presence of rotifers. Moreover, the presence of microplastics does not affect growth and fecundity of the rotifers. This confirms that there are complex and intricate relationships between organisms in the environment that influence the interaction between ciliates and MPs (Scherer et al. 2018).

Interesting results confirming the relationship between preferred food particle size and the size of planktonic ciliates were found by Kivi and Setälä (1995). In their experiments, the authors observed that each of the nine species tested had a specific particle size preference pattern. For example, the optimal particle size varied from 1.4 µm for *Strombidium* sp., which is 20 µm in size, to 9.8 µm for *Strobilidium* sp., which is 40 µm in size. The authors conclude that most ciliate species were able to effectively ingest nanoflagellate-sized prey, but only two of the ciliates studied showed effective grazing on the smallest particles, suggesting a possible ability to utilise bacteria-sized prey (Kivi and Setälä 1995).

Interestingly, it was also observed that the grazing rates of *Euplotes* sp. and *S. teres* were significantly correlated with their cell size. In the case of *Euplotes* sp., the larger the cell, the lower the grazing rate, whereas the relationship between ingestion rate and *S. teres* cell size was positive. These contradictory results may be due to differences in the structure of the oral apparatus as well as differences in locomotion. The clearance rate was not significantly dependent on the cell size of *B. japonicum*. These results differ slightly from those of Fenchel (1980), who reported that the clearance rates of *Euplotes* or *Blepharisma* were dependent on cell volume.

## Conclusions

Our results suggest that MPs are readily ingested by ciliates and could pose a threat to aquatic organisms such as some ciliate species and other zooplankton taxa and that these effects are pronounced when MP concentrations exceed those found in the natural environment, which are e.g. 22.0 ± 0.4 particles m^−3^ on average along the Biobío river basin located in Chile (Correa-Araneda et al. 2022) or 100,000 particles m^−3^ in the Dutch river delta and Amsterdam canals (Leslie et al. 2017). In addition, the ingestion of MPs depends on exposure time, but longer times do not always result in higher ingestion of MP particles. Furthermore, ingestion was also influenced by the diameter of the particles and the size of the ciliate. In our study, ciliates showed a preference for particles with a diameter of 1 µm over 2 µm. Additionally, the highest clearance rates were observed at the lowest concentrations and exposure times. The grazing rate was higher for smaller particles than for larger MPs and generally decreased with increasing incubation time.

This work provides new insights into the study of zooplankton communities and their very important members, ciliates. We have obtained satisfactory results showing that MPs are a serious problem that needs to be investigated in more detail. These results are a further step towards understanding the complex nature of MP pollution in the environment.

## Data Availability

Data available under reasonable request.
